# Task sharing and teamwork: clinician preferences for alcohol screening and brief interventions in cardiology

**DOI:** 10.1186/s13104-025-07452-4

**Published:** 2025-08-29

**Authors:** Paul Welfordsson, Anna-Karin Danielsson, Caroline Björck, Bartosz Grzymala-Lubanski, Kristina Hambraeus, Ida Haugen Löfman, Frieder Braunschweig, Matthias Lidin, Sara Wallhed Finn

**Affiliations:** 1 Department of Global Public Health, Karolinska Institutet, Solna, Sweden; 2 Department of Women’s and Children’s Health, Uppsala University, Akademiska sjukhuset, Uppsala, Sweden; 3 Centre for Research and Development, Region Gävleborg, Gävle, Sweden; 4 Department of Public Health and Clinical Medicine, Umeå University, Umeå, Sweden; 5 Department of Cardiology, Falun Hospital, Falun, Dalarna Sweden; 6 Department of Medicine, Unit of Cardiology, Karolinska Institutet, Stockholm, Sweden; 7 Department of Cardiology, Heart and Vascular Center, Karolinska University Hospital, Stockholm, Sweden; 8 Unit of Clinical Alcohol Research, Institute of Clinical Research, University of Southern Denmark, Odense, Denmark; 9 Centre for Dependency Disorders, Stockholm, Sweden

**Keywords:** Cardiology, Alcohol, Cross-sectional survey, Implementation, Screening, Brief interventions, Preferences

## Abstract

**Objective:**

To investigate clinicians’ preferences for alcohol screening and brief interventions in clinical cardiology settings.

**Results:**

A total of 664 cardiology clinicians responded to a cross-sectional survey (30.9% response rate), including 55.1% nurses, 21.4% assistant nurses, 18.8% doctors, and 4.7% other clinical staff. Among these participants, 87.5% indicated that patients should be screened for alcohol use on cardiology wards, 79.8% in outpatient clinics, 49.1% in emergency departments, and 45.9% on coronary care units. Doctors and nurses were the preferred professions to be responsible for screening across all clinical contexts, while fewer respondents indicated that assistant nurses or physiotherapists should be responsible for screening (*p* < .001). Most participants (85.2%) indicated that patients should be screened in more than one clinical context and 84.6% indicated that more than one profession should be responsible for alcohol screening. Clinicians’ preferred modality for assessing alcohol use was verbal screening (92% of participants), followed by questionnaires (53.5%), digital tools (28.5%), and alcohol biomarkers (22.1%, *p* < .001). Just over half of participants (58%) indicated that they would like to attend training on brief interventions. Findings suggest that task sharing, teamwork, and training may be effective strategies for implementation of alcohol screening and brief interventions in clinical cardiology.

## Introduction

Alcohol screening and brief interventions (BIs) are evidence-based strategies to reduce alcohol-related harm [1]. However, despite evidence indicating effectiveness in general medical settings [2, 3], screening and BIs (SBI) are implemented inconsistently in clinical settings such as cardiology [3–6]. Work is needed to bridge this evidence-practice gap.

Traditionally, alcohol screening has relied on self-report instruments, such as the Alcohol Use Disorders Identification Test (AUDIT) questionnaire [7]. However, our recent studies suggest that clinicians may be sceptical about the reliability of self-reported alcohol use [8, 9], and that such doubts may undermine alcohol prevention practices. Biomarker-based alcohol screening methods, such as phosphatidylethanol (PEth) [10], have emerged as a possible addition, or alternative, to self-report [11]. While laboratory costs and other factors may be barriers to implementation in some settings [12], PEth is a promising screening tool [13]. Positive experiences with PEth have been reported among general practitioners [14]. However, little is known about clinicians’ attitudes and preferences regarding alcohol screening practices in cardiology.

Patients who screen positive for hazardous alcohol use – a pattern of drinking that increases the risks of alcohol-related harm and which affects about one in 12 cardiology patients in Sweden [15] – should be offered BIs [16]. These involve tailored feedback and motivational interviewing, or brief advice to cut down. However, research suggests that many staff members lack competence with BIs [8, 9, 17], and that perceived stigma and low self-efficacy are important barriers to implementation of SBI in cardiology [18, 19]. Sharing SBI tasks among the multi-disciplinary team is suggested as a possible implementation strategy [20], but must reflect clinicians’ views on which professions should implement SBI. Digital interventions are also emerging as promising tools to navigate barriers to SBI [21], yet it is not known whether clinicians perceive these methods to be appropriate in cardiology practice.

This research note extends the findings of our recent mixed methods study which explored clinicians’ perspectives on the feasibility of implementing SBI in cardiology services [8, 19]. The objective of this study was to investigate clinicians’ preferences for SBI in cardiology settings.

## Main text

### Methods

This multi-site cross-sectional study was conducted with approval from the Swedish Ethical Review Authority (2021-06819-01). All participants provided informed consent. Participants were clinical staff (doctors, nurses, assistant nurses, and allied health professionals) working at hospital-based cardiology units in 12 regions across Sweden (Dalarna, Gävleborg, Norrbotten, Skåne, Stockholm, Uppsala, Värmland, Västerbotten, Västra Götaland, Västmanland, Örebro, Östergötland). Recruitment involved contacting unit managers, who forwarded a standardized email, plus two, weekly reminders, to all clinical staff in their cardiology service. This email included study information and a link to a web questionnaire.

We developed a bespoke questionnaire based on findings from our recent qualitative study [8, 19], using REDCap electronic data capture tools [22]. The questionnaire explored clinicians’ preferences for SBI and took ≤ 5 min to complete. Survey data was collected between January–November 2024.

### Variables

#### Profession responsible for screening

Participants were asked to indicate which clinical contexts cardiology patients should be screened in, choosing one or more of: emergency department, coronary care unit, cardiology ward, outpatient clinic. Respondents were then asked to specify which profession(s) should be responsible for screening in each of the clinical contexts specified, choosing one or more of: physiotherapist, doctor, nurse, assistant nurse, other.

#### Preferred screening modalities

Respondents indicated their views on how patients should be asked about their alcohol habits, choosing one or more of: verbally by clinical staff, paper-based questionnaire, digitally, or in conjunction with an alcohol biomarker.

#### Training preferences

All participants were asked whether they would like to attend training on BIs. Those who indicated that they would like to attend training were subsequently asked whether they would prefer training on BIs ’in-person’ or online. A ‘no preference’ option was also provided.

### Covariates

**Hospital type** was categorized as district general or university. Participants self-reported their **gender** (man, woman, or other), **age group** (18–29, 30–39, 40–49, 50–59, ≥ 60 years), **profession** (physiotherapist, doctor, nurse, assistant nurse, other); years of **experience** since qualification (≤ 3 years, 4–10 years, ≥ 10 years), **specialist experience** (ischaemic heart disease, arrhythmia, heart failure, other, no specialist experience), and clinical **work setting** (inpatients = all staff not working in outpatients or in lab/intervention units; outpatients = all staff working in outpatient services, except those also working in lab/intervention units; lab/intervention unit = all staff working in intervention units, regardless of whether they also worked in another setting).

### Statistical methods

We calculated frequencies, percentages, and counts for the variables of interest and assessed these for differences using chi-square, McNemar, Friedman, or Cochran’s Q tests, as appropriate. We examined associations between study covariates (hospital type, gender, age group, profession, experience level, specialist experience, work setting) and participants’ alcohol screening preferences via logistic regression. Our multivariable model investigated factors associated with indicating a preference for two-or-more professions to share responsibility for alcohol screening, both on cardiology wards and in outpatient clinic contexts. Results were reported as odds ratios (ORs) with 95% confidence intervals (CIs). *P*-values < 0.05 were considered statistically significant. Statistical analyses were conducted in StataSE v17.

## Results

### Participants

2146 clinicians were contacted by cardiology unit managers, 693 (32.3%) responded to the survey and 664 (30.9%) completed items associated with this report. Most participants were based in Stockholm (29.2%), followed by Gothenburg (15.3%). Mean age was 45.1 years (range = 20–74). About 75% were women and most (55.1%) were nurses, while 21.4% were assistant nurses, 18.8% doctors, and 4.7% other clinical staff. More than half of participants (51.1%) reported having more than 10 years of work experience in cardiology. Almost half (45.8%) worked in inpatient settings, while 36.8% were employed in outpatient cardiology clinics, and 17.5% worked in lab/intervention settings. Nearly half of participating clinicians (46.5%) reported having specialist experience, including ischaemic heart disease (14.9%), heart failure (12.8%), arrhythmia (10.4%), and other specialist areas of cardiology (8.4%).

### Staff preferences for alcohol screening

Of 664 participants who responded to questions about **professions responsible for screening**, 581 (87.5%) indicated that cardiology patients should be screened for alcohol use on wards, 530 (79.8%) in outpatient clinics (outpatient clinics vs. wards, *p* = .325), 326 (49.1%) in emergency departments (emergency departments vs. wards, *p* < .001), and 305 (45.9%) on coronary care units (coronary care units vs. wards, *p* < .001) (Table 1). Across these clinical contexts, doctors (range = 90.2–96.0%) and nurses (range = 71.8–90.8%) were participants’ preferred professions to be responsible for screening (doctors vs. nurses, *p* = .238), while fewer respondents indicated that assistant nurses (range = 10.8–24.9%; assistant nurses vs. nurses, *p* < .001) or physiotherapists (range = 8.9–16.4%; physiotherapists vs. nurses, *p* < .001) should be responsible for alcohol screening.

**Table 1:** Staff preferences for alcohol screening: profession responsible for screening, according to clinical context (*N* = 664)

Screening preference	Emergency department	Coronary care unit	Cardiology ward	Cardiology outpatient clinic
Any screening	326 (49.1%)	305 (45.9%)	581 (87.5%)	530 (79.8%)
Screened by: *				
Physiotherapist	29 (8.9%)	50 (16.4%)	91 (15.7%)	57 (10.8%)
Doctor	313 (96.0%)	288 (94.4%)	544 (93.6%)	478 (90.2%)
Nurse	234 (71.8%)	277 (90.8%)	526 (90.5%)	452 (85.3%)
Assistant nurse	68 (20.9%)	76 (24.9%)	136 (23.4%)	57 (10.8%)

*Participants were asked to indicate one or more professions; percentages correspond to totals for ‘any screening’

### Shared responsibility for alcohol screening

Among the 664 respondents, 566 (85.2%) indicated that patients should be screened in more than one clinical context (median = 3, IQR = 2–4) and 562 (84.6%) indicated that more than one profession should be responsible for alcohol screening. Doctors and nurses were the mostly frequently selected combination of professions, across all clinical contexts. Preferred number of professions responsible for screening varied according to clinical setting (Friedman test *p* = .001), with fewer screeners suggested in emergency departments and coronary care units (median = 0, IQR = 0–2) than on cardiology wards (median = 2, IQR = 2–2) and in outpatient clinics (median = 2, IQR = 1–2; Table 2).

**Table 2:** Staff preferences for total number of professions that should screen for alcohol use, by clinical context (*N* = 664)

Number of professions	Emergency department	Coronary care unit	Cardiology ward	Cardiology outpatient clinic
0 professions	338 (50.9%)	359 (54.1%)	83 (12.5%)	134 (20.2%)
1 profession	104 (15.7%)	42 (6.3%)	82 (12.4%)	128 (19.3%)
2 professions	146 (22.0%)	172 (25.9%)	335 (50.5%)	320 (48.2%)
3 professions	56 (8.4%)	59 (8.9%)	111 (16.7%)	52 (7.8%)
4 professions	20 (3.0%)	32 (4.8%)	53 (8.0%)	30 (4.5%)

### Factors associated with preferring shared responsibility for alcohol screening

Multivariable logistic regression analysis indicated an association between level of clinical experience and indicating a preference for interprofessional shared responsibility for screening in both inpatient and outpatient cardiology settings; Participants with less than three years of experience in cardiology were almost twice as likely to indicate that responsibility for alcohol screening should be shared by two or more clinical professions, when compared to participants with more than 10 years of experience (OR = 1.87, 95%CI = 1.08–3.18). Other covariates, including hospital type, gender, age group, profession, specialist experience, and work setting, were not associated with preferring shared responsibility for alcohol screening.

### Preferences for alcohol screening modalities

Verbal screening was participants’ preferred implementation format for identifying alcohol use across all clinical work settings (Table 3). Overall, 611 (92%) of participants indicated that patients should be screened verbally by clinical staff, while 355 (53.5%) responded that paper-based questionnaires should be used, 189 (28.5%) supported digital screening, and 147 (22.1%) indicated that patients should be screened in conjunction with an alcohol biomarker (Cochran’s Q test, *p* < .001). While clinical conversation and questionnaires were the most popular screening modalities, participants who worked in outpatient settings were more open to the use of biomarker-assisted screening (OR = 1.90, 95%CI = 1.27–2.83) and digital screening (OR = 1.41, 95%CI = 0.96–2.05) than staff who worked on inpatient wards.

**Table 3:** Staff preferences for alcohol screening modalities, according to clinical work setting (*N* = 664)

Clinical work setting	Verbal screening	Questionnaire (paper)	Digital screening	Biomarker screening
All				
Yes	611 (92.0%)	355 (53.5%)	189 (28.5%)	147 (22.1%)
No	53 (8.0%)	309 (46.5%)	475 (71.5%)	517 (77.9%)
Inpatient ward				
Yes	273 (89.8%)	164 (54.0%)	74 (24.3%)	55 (18.1%)
No	31 (10.2%)	140 (46.0%)	230 (75.7%)	249 (81.9%)
Outpatient clinic				
Yes	229 (93.9%)	133 (54.5%)	76 (31.2%)	72 (29.5%)
No	15 (6.2%)	111 (45.5%)	168 (68.9%)	172 (70.5%)
Lab/intervention				
Yes	109 (94.0%)	58 (50.0%)	39 (33.6)	20 (17.2%)
No	7 (6.0%)	58 (50.0%)	77 (66.4%)	96 (82.8%)

### Training preferences

Of 650 clinicians who responded to a question about training preferences, 377 (58%) indicated that they would like to attend training on BIs, while 124 (19.1%) were neutral, and 149 (22.9%) did not want training on BIs. Among those who were positive to training, 45.5% indicated a preference towards attending training in-person, 27.4% preferred online training, while 27.1% indicated no preference regarding how training was delivered.

## Discussion

This cross-sectional study complements our recent mixed methods study of perceived feasibility for SBI by in cardiology [9] and contributes to accumulating research on SBI implementation [23–25]. Findings indicate that clinicians prefer responsibility for alcohol screening to be shared across multiple clinical contexts and professions within the multi-disciplinary cardiology team. Doctors and nurses were viewed as particularly well-suited to sharing responsibility for alcohol screening on cardiology wards and in clinic settings. While our mixed methods study found that doctors and clinicians with specialist experience tended to view SBI as more feasible, the current survey suggests that less experienced staff may prefer to share responsibility for alcohol screening. Collectively, these findings highlight opportunities for interdisciplinary cardiology practice and point to task-sharing as a promising strategy for implementation of SBI.

Participants’ preferred implementation format was verbal screening, despite increasing access to alcohol biomarkers within Swedish healthcare and recent evidence that many cardiology clinicians doubt the reliability of self-reported alcohol use [8, 9, 13, 18]. Our 2024 qualitative study raised several possible explanations for clinicians’ preference for verbal alcohol screening [8]. During semi-structured interviews, clinicians emphasized the importance of effective communication, both to overcome challenges associated with quantifying alcohol use and to navigate perceived stigma. We also found that many clinicians lacked knowledge of alternative screening methods such as PEth, and several participants reported limited access to and knowledge of printed questionnaires and digital screening tools – a finding that is consistent with wider reports of a lack of training with SBI [17, 26]. Clinicians highlighted the importance of applying an individualised approach to screening and suggested, for example, that electronic screening methods may be more appropriate with younger patients. This may partly explain our current finding of slightly more positive attitudes towards digital screening among staff who worked in outpatients than in ward settings, where the average age of cardiology patients tends to be lower [27]. Our finding that most clinicians were reluctant to recommend biomarker-assisted screening deviated somewhat from those of a recent study of biomarker screening implementation within primary care in Sweden, which reported that staff advocated adopting PEth as a routine test [14]. A possible explanation is that clinicians’ views on PEth also reflect uncertainty of how to handle possible legal consequences of elevated PEth values obtained during routine healthcare interactions, such as reporting patients’ driving licences to the Swedish Transport Agency [28, 29].

Overall, this study highlights the need to ensure that SBI implementation strategies are consistent with clinicians’ preferences, with an emphasis on verbal communication, shared responsibility across clinical contexts, and teamwork [20]. Our finding that just over half of participants were positive to receiving training on BIs is promising, in light of accumulating evidence that clinically relevant training is an effective SBI implementation strategy [9, 17, 25, 30]. Future studies should focus on the sensitivity, specificity, and practical feasibility of different screening modalities in real-world cardiology settings.

## Limitations

Participation was anonymous and confidential, and we emphasized to clinicians that managers would be unaware of their responses. Nevertheless, a response rate of about 30% introduces the potential for selection bias – staff with lower interest in SBI may have been less likely to complete the questionnaire. We were also unable to report response rates according to profession, since this data was not available. However, our response rate was consistent with other surveys involving clinicians in Sweden [31, 32]. Other limitations included non-probability sampling and the lack of formal validation for our survey instrument.

## Conclusion

This study found that cardiology staff prefer responsibility for alcohol screening to be shared within the interdisciplinary team, particularly between doctors and nurses on cardiology wards and in outpatient clinics. Many clinicians preferred verbal screening to alternative modalities, such as questionnaires, digital tools, and biomarkers. Task sharing, teamwork, and training may be effective strategies for SBI implementation in clinical cardiology.

## Data Availability

The datasets used/analyzed during the current study are available from the corresponding author on reasonable request.
